# Response to neoadjuvant chemotherapy and survival in molecular subtypes of resectable gastric cancer: a post hoc analysis of the D1/D2 and CRITICS trials

**DOI:** 10.1007/s10120-022-01280-2

**Published:** 2022-02-07

**Authors:** Hedde D. Biesma, Tanya T. D. Soeratram, Karolina Sikorska, Irene A. Caspers, Hendrik F. van Essen, Jacqueline M. P. Egthuijsen, Aart Mookhoek, Hanneke W. M. van Laarhoven, Mark I. van Berge Henegouwen, Marianne Nordsmark, Donald L. van der Peet, Fabienne A. R. M. Warmerdam, Maud M. Geenen, Olaf J. L. Loosveld, Johanneke E. A. Portielje, Maartje Los, Daniëlle A. M. Heideman, Elma Meershoek-Klein Kranenbarg, Henk H. Hartgrink, Johanna van Sandick, Marcel Verheij, Cornelis J. H. van de Velde, Annemieke Cats, Bauke Ylstra, Nicole C. T. van Grieken

**Affiliations:** 1 Department of Pathology, Cancer Center Amsterdam, Amsterdam University Medical Centers, VU University, De Boelelaan 1117, 1081 HV Amsterdam, The Netherlands; 2 Department of Biometrics, Netherlands Cancer Institute-Antoni van Leeuwenhoek Hospital, Amsterdam, The Netherlands; 3 Department of Gastrointestinal Oncology, Netherlands Cancer Institute-Antoni van Leeuwenhoek Hospital, Amsterdam, The Netherlands; 4 Department of Medical Oncology, Cancer Center Amsterdam, Amsterdam University Medical Centers, University of Amsterdam, Amsterdam, The Netherlands; 5 Department of Surgery, Amsterdam University Medical Centers, University of Amsterdam, Amsterdam, The Netherlands; 6 Department of Oncology, Aarhus University Hospital, Aarhus, Denmark; 7 Department of Surgery, Amsterdam University Medical Centers, VU University, Amsterdam, The Netherlands; 8 Department of Medical Oncology, Zuyderland Hospital, Sittard, The Netherlands; 9 Department of Medical Oncology, OLVG, Amsterdam, The Netherlands; 10 Department of Medical Oncology, Amphia Hospital, Breda, The Netherlands; 11 Department of Medical Oncology, Leiden University Medical Center, Leiden, The Netherlands; 12 Department of Medical Oncology, St. Antonius Hospital, Nieuwegein, The Netherlands; 13 Department of Surgery, Leiden University Medical Center, Leiden, The Netherlands; 14 Department of Surgery, Netherlands Cancer Institute-Antoni van Leeuwenhoek Hospital, Amsterdam, The Netherlands; 15 Department of Radiation Oncology, Netherlands Cancer Institute-Antoni van Leeuwenhoek Hospital, Amsterdam, The Netherlands

**Keywords:** Epstein–Barr virus (EBV), Stomach neoplasms, Histopathological response, Microsatellite instability (MSI), Mucinous differentiation

## Abstract

**Background:**

Epstein–Barr virus positivity (EBV+) and microsatellite instability (MSI-high) are positive prognostic factors for survival in resectable gastric cancer (GC). However, benefit of perioperative treatment in patients with MSI-high tumors remains topic of discussion. Here, we present the clinicopathological outcomes of patients with EBV+, MSI-high, and EBV−/MSS GCs who received either surgery only or perioperative treatment.

**Methods:**

EBV and MSI status were determined on tumor samples collected from 447 patients treated with surgery only in the D1/D2 trial, and from 451 patients treated perioperatively in the CRITICS trial. Results were correlated to histopathological response, morphological tumor characteristics, and survival.

**Results:**

In the D1/D2 trial, 5-year cancer-related survival was 65.2% in 47 patients with EBV+, 56.7% in 47 patients with MSI-high, and 47.6% in 353 patients with EBV−/MSS tumors. In the CRITICS trial, 5-year cancer-related survival was 69.8% in 25 patients with EBV+, 51.7% in 27 patients with MSI-high, and 38.6% in 402 patients with EBV−/MSS tumors. Interestingly, all three MSI-high tumors with moderate to complete histopathological response (3/27, 11.1%) had substantial mucinous differentiation. No EBV+ tumors had a mucinous phenotype. 115/402 (28.6%) of EBV−/MSS tumors had moderate to complete histopathological response, of which 23/115 (20.0%) had a mucinous phenotype.

**Conclusions:**

In resectable GC, MSI-high had favorable outcome compared to EBV−/MSS, both in patients treated with surgery only, and in those treated with perioperative chemo(radio)therapy. Substantial histopathological response was restricted to mucinous MSI-high tumors. The mucinous phenotype might be a relevant parameter in future clinical trials for MSI-high patients.

**Supplementary Information:**

The online version contains supplementary material available at 10.1007/s10120-022-01280-2.

## Background

Gastric cancer (GC) is the fifth most common type of cancer and third among cancer related deaths worldwide [1]. Despite extensive multimodality treatment regimens, patient outcome remains poor [2–4]. Currently, patients with resectable GC are treated with perioperative chemotherapy regimens in Europe [5]. The MAGIC trial showed a statistical significant survival benefit of epirubicin, cisplatin, and 5-fluorouracil (ECF) chemotherapy compared to surgery alone [4]. Recently, the FLOT4 trial showed that 5-fluorouracil (5-FU), leucovorin, oxaliplatin, and docetaxel (FLOT) chemotherapy increased histopathological response and overall survival (OS) [2]. However, benefit of these systemic treatment regimens are highly variable among GC patients [6].

Differences in the histological and molecular composition of GCs can partly explain the differences in biological and clinical behavior of these tumors [6, 7]. Patients with intestinal type tumors are known to have favorable outcome compared to those with diffuse type tumors [8, 9]. Patients with Epstein–Barr virus positive (EBV+) or microsatellite instable (MSI-high) GCs, which are two distinct molecular subtypes within The Cancer Genome Atlas (TCGA) classification, are known to have favorable outcome compared to those with EBV negative and microsatellite stable (EBV−/MSS) GCs [7, 10–13].

Benefit of perioperative chemotherapy in MSI-high GC remains controversial, due to the limited number of these patients in various clinical studies. In the MAGIC trial MSI-high has been suggested as a negative predictive marker for chemotherapy efficacy, which was underlined by lack of histopathological response observed in all nine MSI-high GC patients treated with neoadjuvant chemotherapy [14]. In contrast, in two retrospective series histopathological response to neoadjuvant treatment was seen in four out of 15 and two out of 12 MSI-high tumors [10, 15].

Data on the response rate to neoadjuvant chemotherapy in EBV+ resectable GC are even more limited [16]. One retrospective series showed better OS for seven EBV+ GCs after primary resection compared to five treated with neoadjuvant chemotherapy [10].

Here, we evaluated the histological and molecular composition of tissue samples from the D1/D2 trial, in which patients were treated with surgery only, and the CRITICS trial, in which patients received surgery and perioperative treatment [3, 17]. We hypothesize that only a limited proportion of patients with MSI-high and potentially also EBV+ GC experience substantial benefit from neoadjuvant chemotherapy. The aim of this study was to evaluate differences in histopathological response to neoadjuvant chemotherapy and survival between molecular subtypes of GC.

## Materials and methods

### Patients

Patients who participated in the D1/D2 trial or CRITICS trial (NCT00407186) were included in this study [3, 17]. In the D1/D2 trial, 1078 patients with resectable GC patients were randomized between gastrectomy with limited (D1) and more extended (D2) lymphadenectomy in the Netherlands [17]. None of the patients received any systemic treatment. In the current study, only patients that received the allocated surgical intervention (*N* = 711) were included from the D1/D2 trial. In the CRITICS trial, 788 patients with resectable adenocarcinoma of the stomach or gastro-esophageal junction were randomized between chemotherapy and chemoradiotherapy after preoperative chemotherapy (ECC) and D1+ surgery in the Netherlands, Denmark and Sweden [3]. In the current study, only Dutch patients (*N* = 631) were included from the CRITICS trial. Both arms of the D1/D2 trial were combined and considered the surgery only cohort, and both arms of the CRITICS trial were combined and considered the perioperatively treated cohort, irrespectively of the actual (amount of) treatment administered.

### Materials

Formalin-fixed paraffin-embedded (FFPE) tumor tissues were collected. Resection specimens were collected from the D1/D2 trial [17]. Pre-treatment biopsies as well as resection specimens were collected from the CRITICS trial [3]. Flowcharts of the tumors collected in this study from each trial can be found in Supplementary Figures S1A and S1B.

### Histopathological assessment

The pathology protocol in the D1/D2 trial did not include a minimal number of sections to be taken from the tumor. For this study one tissue block and corresponding slide involving the deepest tumor invasion was available. In the CRITICS trial at least two sections were taken from the primary tumor, including the deepest invasion and areas with suspected serosal involvement. The median number of slides from the tumor or tumor bed, however, was 9 (range 2–38) for 361 patients that underwent resection. Central pathology review of all original slides was performed by a dedicated GI pathologist who determined histological tumor type according to Lauren’s classification and, in case of the CRITICS trial, tumor regression grade (TRG) to preoperative chemotherapy according to Mandard (Table 1) [6, 9, 18]. Tumors that could not be classified according to Lauren were classified as ‘other’, which included tumors with lymphoid stroma, undifferentiated tumors, and mucinous tumors [6]. EBV+ and MSI-high tumors with substantial histopathological response (TRG 1–3) were evaluated in more detail for characteristic morphological features.

**Table 1:** Clinicopathological characteristics of EBV+, MSI-high, and EBV−/MSS patients from the D1/D2 and CRITICS trials

Characteristic	D1/D2 trial (*n* = 447)			*P* value^a^	CRITICS trial (*n* = 454)			*P* value^a^
	EBV+ (*n* = 47)	MSI-high (*n* = 47)	EBV−/MSS (*n* = 353)		EBV+ (*n* = 25)	MSI-high (*n* = 27)	EBV−/MSS (*n* = 402)	
Age at diagnosis (year)								
Median age (IQR)	61 (53–68)	70 (66–77)	65 (56–72)	< 0.001	66 (57–70)	66 (61–75)	63 (55–69)	0.01
Sex, *n* (%)								
Male	43 (91.5)	24 (51.1)	188 (53.3)	< 0.001	22 (88.0)	18 (66.7)	269 (66.9)	0.08
Female	4 (8.5)	23 (48.9)	165 (46.7)		3 (12.0)	9 (33.3)	133 (33.1)	
Tumor localization, *n* (%)								
GE-junction	–	–	–	< 0.001	5 (20.0)	4 (14.8)	68 (16.9)	0.02
Proximal	13 (27.7)	0	33 (9.3)		8 (32.0)	5 (18.5)	73 (18.2)	
Middle	27 (57.4)	7 (14.9)	90 (25.5)		11 (44.0)	7 (25.9)	121 (30.1)	
Distal	6 (12.8)	35 (74.5)	188 (53.3)		1 (4.0)	11 (40.7)	140 (34.8)	
> 2/3 of stomach	1 (2.1)	5 (10.6)	42 (11.9)		–	–	–	
Lauren classification, *n* (%)								
Diffuse	6 (12.8)	4 (8.5)	118 (33.4)	< 0.001^b^	4 (16.0)	3 (11.1)	194 (48.3)	< 0.001
Intestinal	30 (63.8)	34 (72.3)	169 (47.9)		13 (52.0)	20 (74.1)	142 (35.3)	
Mixed	1 (2.1)	2 (4.3)	27 (7.6)		2 (8.0)	0	28 (7.0)	
Other	10 (21.3)	7 (14.9)	36 (10.2)		6 (24.0)	4 (14.8)	38 (9.5)	
Missing	0	0	3 (0.8)		–	–	–	
(y)pT stage^c^, *n* (%)								
pT1	13 (27.7)	4 (8.5)	75 (21.1)	0.20^b^	4 (16.0)	1 (3.7)	40 (10.0)	0.56^b^
pT2	3 (6.4)	9 (19.1)	44 (12.5)		3 (12.0)	2 (7.4)	48 (11.9)	
pT3	19 (40.4)	20 (42.6)	133 (37.7)		8 (32.0)	16 (59.3)	153 (38.1)	
pT4	12 (25.5)	14 (29.8)	100 (28.3)		4 (16.0)	5 (18.5)	81 (20.1)	
Missing	0	0	1 (0.3)		6 (24.0)	3 (11.1)	80 (19.9)	
(y)pN stage^c^, *n* (%)								
pN0	29 (61.7)	26 (55.3)	115 (32.6)	< 0.001	16 (64.0)	13 (48.1)	141 (35.1)	0.03^b^
pN1	6 (12.8)	8 (17.0)	70 (19.8)		3 (12.0)	6 (22.2)	56 (13.9)	
pN2	7 (14.9)	7 (14.9)	79 (22.4)		0	3 (11.1)	75 (18.7)	
pN3	5 (10.6)	6 (12.8)	89 (25.2)		3 (12.0)	2 (7.4)	60 (14.9)	
Missing	0	0	0		3 (12.0)	3 (11.1)	70 (17.4)	
TNM (7th edition), *n* (%)								
Stage 0/pCR	–	–	–	0.04	3 (12.0)	0	10 (2.5)	0.03^b^
Stage I	14 (29.8)	10 (21.3)	88 (24.9)		6 (24.0)	3 (11.1)	63 (15.7)	
Stage II	19 (40.4)	21 (44.7)	91 (25.8)		9 (36.0)	15 (55.6)	118 (29.4)	
Stage III	13 (27.7)	15 (31.9)	162 (45.9)		4 (16.0)	6 (22.2)	140 (34.8)	
Stage IV	1 (2.1)	1 (2.1)	12 (3.4)		–	–	–	
Missing	0	0	0		3 (12.0)	3 (11.1)	71 (17.7)	
Histopathological response (Mandard), *n* (%)								
Good (TRG 1 + 2)	–	–	–		5 (20.0)	2 (7.4)	43 (10.7)	0.08^b^
Moderate (TRG 3)	–	–	–		3 (12.0)	1 (3.7)	72 (17.9)	
Poor (TRG 4 + 5 + progression)	–	–	–		13 (52.0)	24 (88.9)	253 (62.9)	
Unknown	–	–	–		4 (16.0	0	34 (8.5)	

The histopathological variables are determined at central pathology review

*EBV* + Epstein-Barr virus positive, *MSI-high* microsatellite instable, *EBV-/MSS* Epstein–Barr virus negative and microsatellite stable, *IQR* interquartile range, *pCR* pathological complete response

^**a**^*P*-values are derived from Fisher’s exact tests between the three groups. ANOVA was used for continuous variable age. Those with missing data are excluded

^b^Excluding those with missing data

^c^yp denotes the T, N, and TNM stages after neoadjuvant chemotherapy and surgery in the CRITICS trial

### EBV analysis

EBV-encoded RNA in situ hybridization (EBER-ISH) was performed to determine EBV status as previously described [13]. For this purpose, tissue microarrays (TMAs) of resection specimens were used. In case tumor areas in the resection specimens were too small to use in TMAs, a four µm whole slide was used. In case no resection specimen was available of a patient from the CRITICS trial, a four µm slide from the pre-treatment biopsy was used. A detailed description of the EBV analysis method can be found in the Supplementary Materials.

### MSI analysis

DNA was extracted as previously described [19]. In brief, the tumor containing area was demarcated by a GI pathologist (NCTvG) on the H&E slide from the surgical resection specimens (D1/D2 trial) or pre-treatment biopsies (CRITICS trial). For the CRITICS trial, surgical resection specimens were used in case no tumor was left in the H&E slide of a biopsy. Demarcated tumor areas were macrodissected on adjacent serial 10 µm slides and genomic DNA was extracted using the QIAamp DNA Micro kit (Qiagen, Westburg, Leusden, The Netherlands). Tumor DNA was analyzed for MSI status with five near-monomorphic mononucleotide markers (BAT-25, BAT-26, MONO-27, NR-21, and NR-24) using a fluorescent multiplex PCR-based method (MSI Analysis System, Promega Corporation, Madison, WI, USA). Tumors were considered MSI-high if at least two out of five mononucleotide markers showed instability, and as MSI-low or MSS (further grouped as MSS) if one or none of the markers examined showed instability. Immunohistochemistry (IHC) for mismatch repair proteins (MLH-1, MSH-2, MSH-6, PMS-2) was performed in case MSI-PCR failed. A detailed description of the MSI analysis method can be found in the Supplementary Materials.

### Statistical analysis

Based on histopathological response to chemotherapy in the CRITICS trial, patients with TRG 1 and 2 tumors were grouped together as ‘good responders’, whereas TRG 3 were considered ‘moderate responders’. Patients who did not undergo surgical resection because of progression of disease while being treated with chemotherapy were grouped as ‘poor responders’, together with patients with TRG 4 and 5 tumors. Patients who did not undergo surgical resection, because of progression before start of chemotherapy, non-GC-related death, or patients’ wish, were not analyzed for histopathological response. Fisher’s exact tests were used to correlate clinicopathological variables with molecular subgroups. One-way ANOVA was used to correlate continuous variables with these subgroups. Log-rank tests were used to correlate EBV and MSI status with survival. Kaplan–Meier plots were ended when < 10% of patients per subgroup were at risk. OS was defined as time from randomization till death by any cause. Cancer-related survival (CRS) was defined as time from randomization till recurrence or progression of disease, or death related to GC. A Cox model was used to calculate Hazard Ratios (HRs). EBV and MSI status as well as variables with *P* < 0.10 from univariable analyses were included in the multivariable Cox model. All analyses were conducted using the program language R (version 3.6.1).

## Results

### EBV and MSI status

Sufficient tumor tissue for both EBV and MSI analyses was available from 447/711 (62.9%) patients in the D1/D2 trial, and 454/631 (71.9%) Dutch patients in the CRITICS trial (Supplementary Figures S1A and S1B). The majority of EBV+ samples in the D1/D2 trial has been described before [13].

**Fig. 1:**
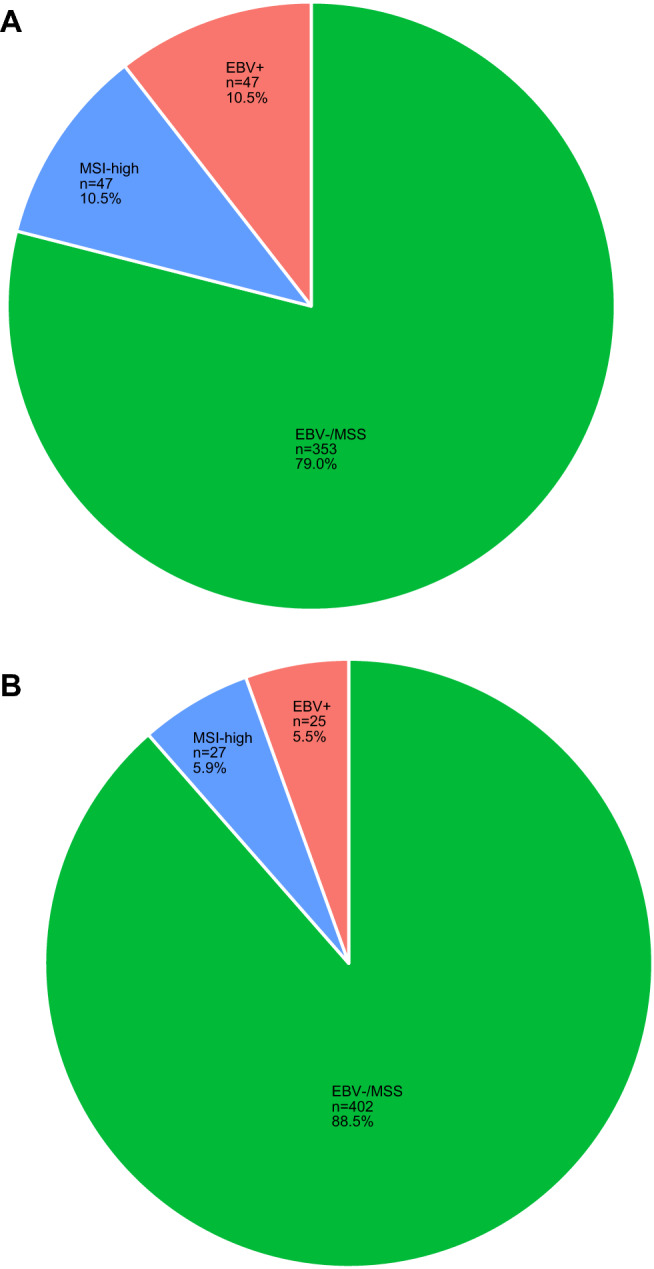
Molecular subgroups of gastric cancer in patients from **a** the D1/D2 and **b** CRITICS trials. Numbers and proportions of the molecular subgroups EBV+, MSI-high, and EBV−/MSS are indicated and mutually exclusive for both cohorts. *EBV*+ Epstein–Barr virus positive, *MSI-high* microsatellite instable, *EBV−/MSS* Epstein–Barr virus negative and microsatellite stable

In the D1/D2 trial, MSI analysis was successfully performed by PCR in 414/447 (92.6%) cases. IHC was performed in 33/447 (7.4%) cases with missing PCR data, of which 32 had proficient mismatch repair (pMMR) proteins and were assigned to the MSS group, and one had deficient MMR proteins (dMMR) and was assigned to the MSI-high group. This resulted in 47 (10.5%) EBV+ and 47 (10.5%) MSI-high cases in the D1/D2 trial. EBV+ and MSI-high were mutually exclusive (Fig. 1A).

In the CRITICS trial, MSI analysis was performed on 351 biopsies and 103 surgical resection specimens. MSI status was successfully obtained by PCR in 423/454 (93.2%) cases. IHC was performed in 31/454 (6.8%) cases with missing PCR data, all 31 had pMMR proteins, and were therefore grouped with MSS. EBER-ISH was performed on TMA cores of resection specimens in 216/454 (47.6%), whole slides from resection specimens in 81/454 (17.8%), and diagnostic biopsy specimens in 157/454 (34.6%) cases. This resulted in 25 (5.5%) EBV+ and 27 (5.9%) MSI-high cases in the CRITICS. Again, EBV+ and MSI-high were mutually exclusive (Fig. 1B).

### Clinicopathological characteristics

No differences in clinicopathological characteristics were found between the patients from whom tumor tissue was available for further analyses and both original trial populations (Supplementary Tables S1A and S1B). The patients in the current analyses showed similar survival as the total trial populations (Supplementary Figs. S2A and S2B).

Clinicopathological characteristics per molecular subgroup are shown in Table 1. Histopathological data were obtained after central pathology review. EBV+ tumors occurred more often in male patients, compared to EBV−/MSS tumors (*P* < 0.001 in D1/D2, and *P* = 0.03 in CRITICS). They were most often localized in the upper part of the stomach, whereas EBV−/MSS tumors were mostly found in the distal part of the stomach (*P* < 0.001 in D1/D2, and *P* = 0.003 in CRITICS). EBV+ tumors had less lymph node involvement than EBV−/MSS tumors (*P* = 0.002 in D1/D2, and *P* = 0.01 in CRITICS).

Patients with MSI-high tumors were older than patients with EBV−/MSS tumors (*P* < 0.001 in D1/D2, and *P* = 0.003 in CRITICS). MSI-high tumors were most often located in the distal part of the stomach in the D1/D2 trial (*P* = 0.01), but this did not reach significance in the CRITICS trial. Both EBV+ and MSI-high tumors were more often of the intestinal histological subtype, compared to EBV-/MSS tumors in the CRITICS trial (*P* = 0.003 and *P* < 0.001, respectively), but this did not reach significance in the D1/D2 trial. MSI-high tumors were only associated with low lymph node stage in the D1/D2 trial (*P* = 0.02), but this did not reach significance in the CRITICS trial. Although not statistically significant, EBV+ GCs had the highest (near-)complete histopathological response (TRG 1–2) rate: EBV+ (22.7%, 5/22), MSI-high (7.4%, 2/27), and EBV−/MSS (11.3%, 43/382) tumors. TRG per molecular subgroup is shown in Supplementary Fig. S3.

### Survival

**Fig. 2:**
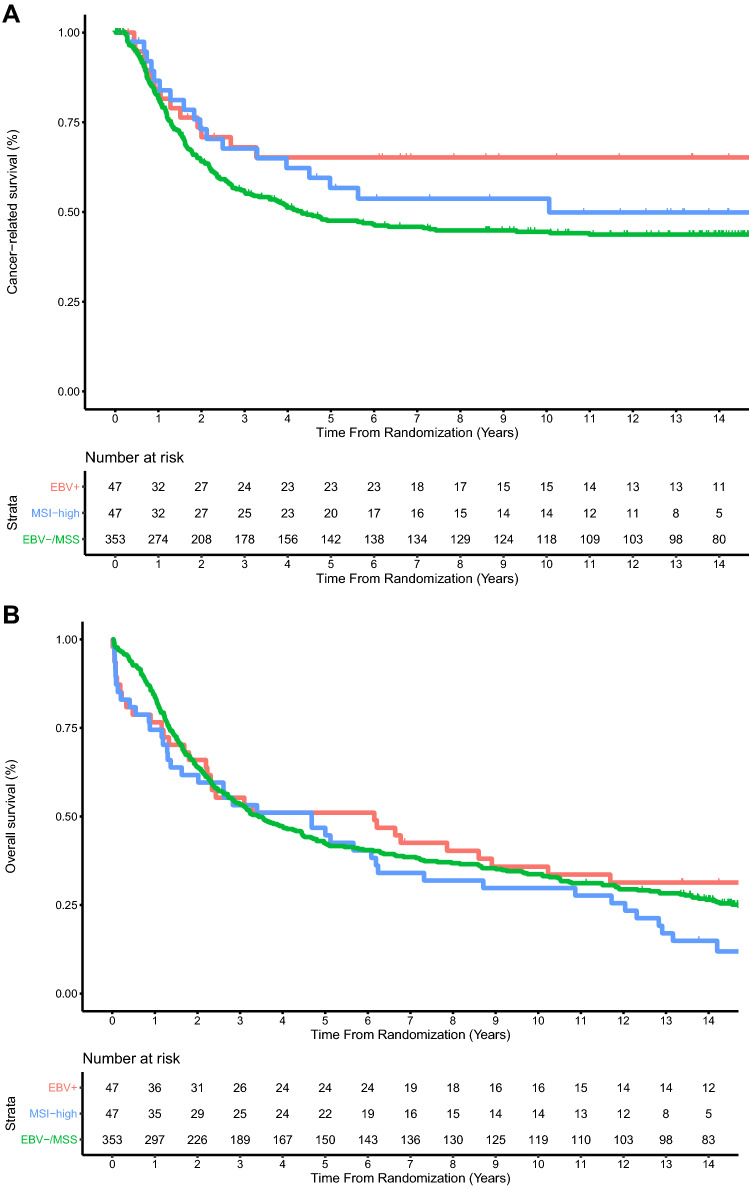
**a** Cancer-related and **b** overall survival since randomization in 447 patients of the Dutch D1/D2 trial. **a** The hazard ratio was 0.57 (95% CI 0.31–0.99, *P* = 0.047) for EBV+ vs EBV−/MSS, and 0.78 (95% CI 0.48–1.27, *P* = 0.32) for MSI-high vs EBV−/MSS. **b** The hazard ratio was 0.90 (95% CI 0.63–1.30, *P* = 0.59) for EBV+ vs EBV−/MSS, and 1.31 (95% CI 0.92–1.82, *P* = 0.10) for MSI-high vs EBV−/MSS. *EBV*+ Epstein–Barr virus positive, *MSI-high* microsatellite instable, *EBV−/MSS* Epstein–Barr virus negative and microsatellite stable

In the D1/D2 trial 5-year CRS was 65.2% for EBV+, 56.7% for MSI-high, and 47.6% for EBV−/MSS (HR 0.57, 95% CI 0.31–0.99, *P* = 0.047 for EBV+, and HR 0.78, 95% CI 0.48–1.27, *P* = 0.32 for MSI-high, both compared to EBV−/MSS; Fig. 2A). Five-year OS was 51.1% for EBV+, 46.8% for MSI-high, and 42.5% for EBV−/MSS (HR 0.90, 95% CI 0.63–1.30, *P* = 0.59 for EBV+, and HR 1.31, 95% CI 0.92–1.82, *P* = 0.10 for MSI-high, both compared to EBV−/MSS; Fig. 2B).

**Fig. 3:**
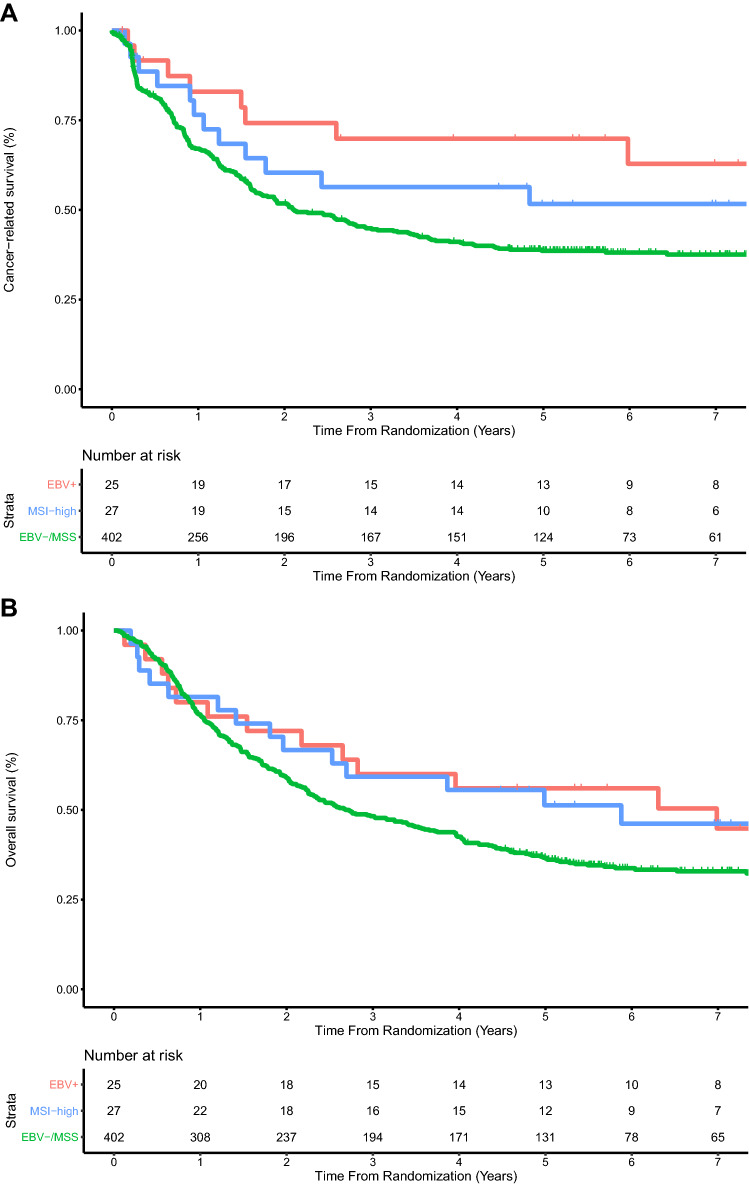
**a** Cancer-related and **b** overall survival since randomization in 454 patients of the CRITICS trial. **a** The hazard ratio was 0.44 (95% CI 0.22–0.88, *P* = 0.02) for EBV+ vs EBV-/MSS, and 0.67 (95% CI 0.37–1.19, *P* = 0.17) for MSI-high vs EBV−/MSS. **b** The hazard ratio was 0.64 (95% CI 0.36–1.11, *P* = 0.11) for EBV+ vs EBV−/MSS, and 0.67 (95% CI 0.39–1.14, *P* = 0.14) for MSI-high vs EBV−/MSS. *EBV*+ Epstein–Barr virus positive, *MSI-high* microsatellite instable, *EBV−/MSS* Epstein–Barr virus negative and microsatellite stable

In the CRITICS trial 5-year CRS was 69.8% for EBV+, 51.7% for MSI-high, and 38.6% for EBV−/MSS (HR 0.44, 95% CI 0.22–0.88, *P* = 0.02 for EBV+, and HR 0.67, 95% CI 0.37–1.19, *P* = 0.17 for MSI-high, both compared to EBV−/MSS; Fig. 3A). Five-year OS was 56.0% for EBV+, 51.3% for MSI-high, and 36.7% for EBV−/MSS (HR 0.64, 95% CI 0.36–1.11, *P* = 0.11 for EBV+, and HR 0.67, 95% CI 0.39–1.14, *P* = 0.14 for MSI-high, both compared to EBV−/MSS; Fig. 3B).

Based on univariate analyses, the multivariate Cox model on the molecular subgroups for each trial included age, sex, tumor localization, histological tumor type, and TNM stage. In the D1/D2 trial the overall model resulted in HR 0.86, 95% CI 0.47–1.59, *P* = 0.64 for EBV+, and HR 0.90, 95% CI 0.54–1.51, *P* = 0.70 for MSI-high, both compared to EBV−/MSS. In the CRITICS trial the overall model also included the different postoperative treatment regimens and resulted in HR 0.91, 95% CI 0.44–1.87, *P* = 0.79 for EBV+, and HR 0.76, 95% CI 0.41–1.40, *P* = 0.38 for MSI-high, both compared to EBV−/MSS. The statistical significant prognostic factors for survival in both trials were age and TNM stage.

### Mucinous differentiation and histological response in MSI-high tumors

**Fig. 4:**
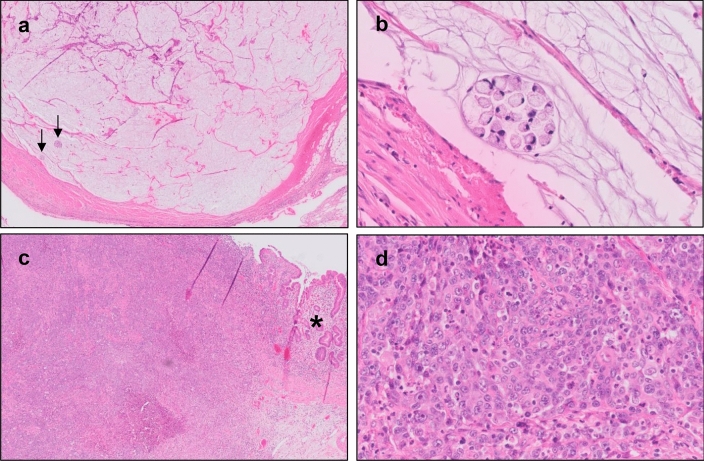
Histopathological response in MSI-high carcinomas after neoadjuvant chemotherapy. **a** Microscopic image of a resection specimen of a MSI-high mucinous adenocarcinoma with near-complete histopathological response (TRG 2) showing huge mucinous lakes with only focal presence of few remaining vital tumor cells (arrows). **b** Detail of a cluster of vital tumor cells with signet ring cell morphology. **c** Resection specimen of a MSI-high adenocarcinoma with a solid growth pattern in relation to normal mucosa (asterisk) with no signs of histopathological response (TRG 5). **d** Detail of the tumor shows the solid growth pattern with no glandular structures or mucinous differentiation

In the CRITICS trial, two MSI-high tumors showed (near-)complete histopathological response (TRG 1–2). They were both classified as ‘other’ according to Lauren and as mucinous according to the WHO 7th edition classification [20] with > 50% of the tumor showing mucinous differentiation (Fig. 4A, B) with (almost) no vital tumors cells present. In addition, the only MSI-high tumor with moderate response (TRG 3) showed several mucinous lakes without tumor cells (25–50%), but also a large area with tubular architecture without signs of histopathological response, and was classified as intestinal by Lauren’s classification. Of the 20 MSI-high evaluable tumors with little or no sign of histopathological response (TRG 4–5) some mucinous lakes (< 25% of the total tumor area) were present in four cases, of which two contained tumor cells in these lakes. Areas of fibrosis, the most frequent sign of histopathological response, were not seen in MSI-high tumors (Fig. 4C, D). Hence, histopathological response to chemotherapy was only seen in the mucinous parts of the MSI-high tumors.

Five EBV+ tumors showed (near-)complete histopathological response (TRG 1–2), but none of them contained mucinous lakes. In EBV−/MSS tumors with TRG 1–2, mucinous lakes were observed in 9/36 (25.0%). No correlation between TRG and mucinous differentiation was observed in EBV−/MSS tumors.

In the D1/D2 trial, mucinous lakes were observed in 27 out of 385 cases, including six MSI-high tumors. All 27 tumors showed presence of vital tumor cells within these mucinous areas.

### Mucinous differentiation in pre-treatment biopsies

Detection of mucinous differentiation in pre-treatment biopsies would be clinically relevant, since treatment decisions for MSI-high GC patients will have to be made on these biopsies. Corresponding biopsies and resection slides were available of 26 tumors (*N* = 6 MSI-high and *N* = 20 EBV-/MSS) with mucinous lakes in the CRITICS trial. The presence of mucinous differentiation in biopsy specimens was correlated to the percentage (in quartiles) of the tumor area that consisted of mucinous lakes in the resection specimens: 1–25%, 26–50%, 51–75% and 76–100%. A mucinous phenotype (> 50% of resection specimen consisting of mucinous lakes) could be reliably detected in 13/15 pre-treatment biopsies (Supplementary Tables S2A and S2B). A higher percentage of mucinous lakes in resection specimens was correlated to a higher chance of mucinous lakes found in biopsies (*P* = 0.07). Supplementary Fig. S4 shows an example of a pre-treatment biopsy of a MSI-high mucinous GC.

## Discussion

There is an ongoing debate on benefit of chemotherapy for MSI-high GC [10, 14, 15, 21, 22]. Our study shows that patients with MSI-high GCs have a numerically prolonged cancer-related survival compared to EBV−/MSS GCs regardless of whether they were treated with surgery alone or in combination with perioperative chemo(radio)therapy. This is in contrast with the MAGIC trial, in which the survival for patients with MSI-high GC treated with chemotherapy was detrimental [14]. In addition, we identified two (7.4%) MSI-high tumors with a good histopathological response to chemotherapy (TRG 1–2) and one (3.7%) with moderate histopathological response (TRG 3), while no good responders were identified in the MAGIC trial [14]. The absence of histopathological responders in the MAGIC trial might be explained by the small number of MSI-high GCs (*N* = 9) in the chemotherapy arm.

Interestingly, the two MSI-high GCs with good histopathological response were both classified as mucinous carcinomas according to the WHO classification. The MSI-high tumor with TRG 3 also consisted for large parts of mucinous lakes, although just less than 50% of the total tumor area. The mucinous lakes in these three MSI-high tumors did not contain any tumor cells. None of the MSI-high tumors without mucinous differentiation showed a substantial histopathological response. In the D1/D2 trial all mucinous GCs showed presence of tumors cells in the mucinous lakes, suggesting that the lack of tumor cells in mucinous lakes is indeed a sign of response to chemotherapy. Hence, these findings suggest that chemotherapy for MSI-high tumors might be effective only in patients with tumors that consist for a large part of mucinous lakes. However, the rationale behind this remains unclear. One hypothesis would be that chemotherapy could better reach tumor cells when they reside within or around a mucinous lake. This mechanism of action has not been proven yet, although there are reports that tumor cells within or around mucinous lakes do respond to chemotherapy, such as in mucinous breast and colorectal cancer [23–25]. A relation between chemotherapy and a mucinous phenotype is not unprecedented but results are conflicting. Mucinous breast and colorectal adenocarcinomas that have similar genetic characteristics as gastric cancer [26], have also been associated with poor response to chemotherapy [27–30]. In these studies however no distinction was made between MSI-high and MSS mucinous tumors. Hence, a genetic explanation would be more plausible, such that the genetic makeup of MSI-high mucinous gastric cancer might differ from other mucinous cancers.

The histopathological response in mucinous MSI-high tumors raises the question whether this phenomenon is specific for MSI-high GC, or also present in other molecular subtypes. In our cohort of the CRITICS trial, none of the EBV+ tumors showed mucinous differentiation and 2 out of 27 (7.4%) MSI-high tumors were classified as mucinous, which is in concordance with the TCGA dataset, in which the mucinous subtype was present in none of the EBV+ and 10.4% of the MSI-high tumors [7]. Only 5.4% of EBV−/MSS tumors within TCGA were classified as mucinous [7]. In the EBV−/MSS group of the CRITICS trial only *N* = 13 tumors classified as mucinous carcinomas in the resection specimens had mucinous lakes present in the matched pre-treatment biopsies. Larger cohorts are required to draw reliable conclusions on the effect of chemotherapy on histopathological response and survival in this morphological subgroup of GC. A recent survival analysis of patients with stage I-III GC in the SEER database did not show clinically relevant survival differences between so-called mucin-producing (*N* = 1515) and conventional adenocarcinomas (*N* = 4174), either when they were treated with surgery only or chemotherapy. The study included signet ring cell carcinomas in the mucin-producing group, which may have obscured the survival outcomes of patients with tumors consisting mainly of extracellular mucus. MSI status was not reported in the study [31].

We showed that mucinous differentiation can be found in the pre-treatment biopsy of the vast majority of mucinous carcinomas. This finding requires validation in larger series. However, in a few cases the mucinous differentiation was not found in the biopsy, which can be caused by sampling error, since the biopsy is only a small representation of the whole tumor. Whether chemotherapy changes this mucinous differentiation, or the histological subtype in general, is unclear.

Overall, clinicopathological features associated with EBV+ and MSI-high GC are in line with previous studies, such as the predominance of the intestinal subtype [7, 10, 13, 14, 32–34]. TCGA reported 19.2% diffuse and 57.7% intestinal subtype in EBV+, 9.4% diffuse and 75.0% intestinal subtype in MSI-high, and 28.3% and 64.9% in EBV−/MSS. This high predominance of the intestinal subtype in TCGA was not seen in the D1/D2 and CRITICS trials, which can be explained by a higher prevalence of intestinal subtype in Asian countries [35]. EBV+ and MSI-high GCs occurred in lower percentages in the D1/D2 trial compared to the CRITICS trial. This may be explained by the rising incidence of proximal tumors, whereas EBV+ and MSI-high GCs occur more often in the middle and distal part of the stomach, respectively [36]. Clinicopathological features associated with favorable survival outcomes were lower stage of disease and younger age, irrespective of molecular subgroup. EBV+ tumors showed highest histopathological response rate after neoadjuvant treatment. In addition, the known favorable outcome after surgery alone of EBV+ compared to EBV−/MSS GC remains when treated with perioperative chemotherapy. Therefore, our data do not indicate that chemotherapy increases mortality in EBV+ GCs.

We observed substantial differences in CRS and OS. This could potentially be explained by the relatively old age of onset of GC patients. CRS would better reflect the survival outcome related to tumor biology, whereas cardiovascular and other diseases determine OS in a subset of patients. The difference between CRS and OS was most prominent in the EBV+ subgroup. This could in part be explained by the high proportion of male patients in this subgroup, who in general have a shorter life expectancy than females [37, 38].

Our D1/D2 and CRITICS cohorts are among the largest GC series reported. Nevertheless, our findings are limited by the fact that neither trial included both a surgery only and a perioperative chemotherapy arm. Therefore, direct comparison of survival between patients per molecular subtype would not be reliable. There is a substantial time gap between the two trials and the D1/D2 trial contains a relatively high number of early (pT1N0) tumors. In the CRITICS trial ECC was used as perioperative chemotherapy regimen, while currently perioperative FLOT is the standard of care in Europe. Whether a mucinous phenotype could be important for response to FLOT in MSI-high GC still needs to be evaluated.

In conclusion, among molecular subgroups of GCs EBV+ tumors showed the highest histopathological response rate and favorable outcome compared to EBV−/MSS. We found substantial histopathological response after neoadjuvant chemotherapy in MSI-high GC, but only in those with a mucinous phenotype. These results indicate that the mucinous phenotype might be a relevant parameter in future clinical trials for MSI-high patients.

## Supplementary Information

Below is the link to the electronic supplementary material.

Supplementary file1 (PDF 1018 kb)
