# Supplementary material for: Response to neoadjuvant chemotherapy and survival in molecular subtypes of resectable gastric cancer: a post hoc analysis of the D1/D2 and CRITICS trials
Source: Gastric Cancer. 2022 Feb 7;25(3):640–51. doi: 10.1007/s10120-022-01280-2 (PMC9013342; doi:10.1007/s10120-022-01280-2)
Supplement: Supplementary file 1 — Supplementary file1 (PDF 1018 kb) [file 10120_2022_1280_MOESM1_ESM.pdf]

Supplementary Materials

Response to neoadjuvant chemotherapy and survival in molecular subtypes of resectable gastric cancer: a post hoc analysis of the D1/D2 and CRITICS trials

H.D. Biesma, T.T.D. Soeratram, K. Sikorska, I.A. Caspers, H.F. van Essen, J.M.P. Egthuijsen, A. Mookhoek, H.W.M. van Laarhoven, M.I. van Berge Henegouwen, M. Nordsmark, D.L. van der Peet, F.A.R.M. Warmerdam, M.M. Geenen, O.J.L. Loosveld, J.E.A. Portielje, M. Los, D.A.M. Heideman, E. Meershoek-Klein Kranenbarg, H.H. Hartgrink, J. van Sandick, M. Verheij, C.J.H. van de Velde, A. Cats, B. Ylstra, and N.C.T. van Grieken.

Supplementary Methods.

A. Ethical approval

B. Detailed EBV methodology

C. Detailed MSI methodology: PCR

D. Detailed MSI methodology: immunohistochemistry

Supplementary Results.

Immunohistochemistry on MSI-high tumors

Supplementary Tables

Supplementary Table S1A. Clinicopathological characteristics of the 447 study patients per treatment arm of the D1/D2 trial.

Supplementary Table S1B. Clinicopathological characteristics of the 454 study patients per treatment arm of the CRITICS trial.

Supplementary Table S2A. Correlation of mucinous lakes found in pre-treatment biopsies and resection specimens in the CRITICS trial.

Supplementary Table S2B. Correlation of mucinous lakes found in pre-treatment biopsies and tumor regression grade of MSI-high tumors in the CRITICS trial.

Supplementary Figures

Supplementary Figure S1A. Flowchart of tumor material from patients in the D1/D2 trial used for EBV and MSI analyses.

Supplementary Figure S1B. Flowchart of tumor material from patients in the CRITICS trial used for EBV and MSI analyses.

Supplementary Figure S2A. Cancer-related and overall survival since randomization in the 447 study patients per treatment arm of the D1/D2 trial.

Supplementary Figure S2B. Cancer-related and overall survival since randomization in the 454 study patients per treatment arm of the CRITICS trial.

Supplementary Figure S3. Histopathological response according to Mandard per molecular subgroup in the CRITICS trial.

Supplementary Figure S4. Biopsy specimen of a MSI-high carcinoma with mucinous differentiation.

Supplementary Methods

A. Ethical approval

The D1/D2 trial was approved by the medical ethical committee of the Leiden University Medical Centre. Since all D1/D2 patients succumbed at the start of this study, the Dutch Code of Conduct for Responsible Use of Human Tissue allows for the analysis of these residual tissue specimens obtained for diagnostic purposes and anonymized publication of the results (https://www.federa.org/sites/default/files/images/print_version_code_of_conduct_english.pdf). The CRITICS trial was approved by the medical ethical committee of the Netherlands Cancer Institute and the review boards of all participating centers. Patients provided written informed consent for participation into the clinical trial and separately for translational research on residual material. The D1/D2 and CRITICS scientific committees approved of the current study.

B. Detailed EBV methodology

Tissue microarrays (TMAs) were constructed from tumor blocks of resection specimens and composed of three 1.0 mm cores per tumor. Non-neoplastic spleen tissue was used as control. Slides were stained by a Ventana automated platform (Ventana Medical Systems Inc., Tucson, AZ, USA) with an EBER probe kit by Ventana and incubated according to the manufacturer's protocol, after previous treatment of the slides with ISH protease 3 for 4 minutes, after which EBER was detected with ISH iVIEW blue. Presence of EBER was scored by a GI pathologist (AM, NCTvG). Any presence of EBER inside the demarcated tumor area was considered EBV+.

C. Detailed MSI methodology: PCR

After deparaffinization with xylene, DNA was extracted by using the column-based method QIAamp DNA micro kit (Qiagen, Westburg, Leusden, the Netherlands). DNA was quantified by Qubit dsDNA broad range assay (Thermo Fisher Scientific Inc., Waltham, MA, USA) according to the manufacturer's

protocol. Tumor DNA was analyzed for MSI with five near-monomorphic mononucleotide markers (BAT-25, BAT-26, MONO-27, NR-21, and NR-24) using a fluorescent multiplex PCR-based method (MSI Analysis System, Version 1.2 , Promega Corporation, Madison, WI, USA) according to manufacturer's protocol. Once amplified, samples were analyzed by capillary electrophoresis using a 3500 Genetic Analyzer (Applied Biosystems, Thermo Fisher Scientific, Inc.). Data were processed with GeneMapper software (Thermo Fisher).

D. Detailed MSI methodology: immunohistochemistry

Immunohistochemistry was performed using a Ventana automated platform with protein detection by Optiview. Clone G168-15 by Becton and Dickinson was used for MLH-1 detection at a dilution of 1/25 and incubated for 64 minutes after previous treatment of the slides with CC1 for 32 minutes. Clone G219-1129 by Monosan was used for MSH-2 detection at a dilution of 1/100 and incubated for 32 minutes after previous treatment of the slides with CC1 for 48 minutes. Clone EP49 by Agilent/Dako was used for MSH-6 detection at a dilution of 1/25 and incubated for 64 minutes after previous treatment of the slides with CC1 for 32 minutes. Clone EPR3947 by Cell Marque Corp. was used for PMS-2 detection at a ready-to-use dilution and incubated for 32 minutes after previous treatment of the slides with CC1 for 64 minutes.

In addition, IHC was performed on all MSI-high tumors to determine which of the mismatch repair genes was dysfunctional.

Supplementary Results. Immunohistochemistry on MSI-high tumors

Sufficient material of 20/47 (42.6%) MSI-high tumors was available to determine which of the mismatch repair genes was dysfunctional. All 20 tumors showed simultaneous protein loss of MLH-1 and PMS-2. One MSI-high tumor had insufficient tumor tissue left for IHC. The majority (19/26, 73.1%) of the remaining MSI-high tumors showed simultaneous protein loss of MLH-1 and PMS-2. One of these 19 cases had an additional loss of MSH-6 expression. Two cases showed protein loss of both MSH-2 and MSH-6, and two cases had an isolated PMS-2 loss. The three remaining MSI-high cases showed no loss of any of these four MMR proteins by IHC.

Both MSI-high tumors with (near-)complete histopathological response in the CRITICS trial showed simultaneous protein loss of MLH-1 and PMS-2.

D1/D2 trial			
**Characteristic**	**D1 surgery (*n* = 234)**	**D2 surgery (*n* = 213)**	***P* value[a]**
Age at diagnosis (year)			
Median age (IQR)	67 (58-73)	64 (54-72)	0.06
Sex, *n* (%)			
Male	125 (53.4)	130 (61.0)	0.13
Female	109 (46.6)	83 (39.0)	
Tumor localization, *n* (%)			
Proximal	25 (10.7)	21 (9.9)	0.81
Middle	69 (29.5)	55 (25.8)	
Distal	116 (49.6)	113 (53.1)	
>2/3 of stomach	24 (10.3)	24 (11.3)	
Lauren classification, *n* (%)			
Diffuse	64 (27.4)	64 (30.0)	0.44[b]
Intestinal	122 (52.1)	111 (52.1)	
Mixed	20 (8.5)	10 (4.7)	
Other	28 (12.0)	25 (11.7)	
Missing	0	3 (1.4)	
pT stage, *n* (%)			
pT1	50 (21.4)	42 (19.7)	0.88[b]
pT2	30 (12.8)	26 (12.2)	
pT3	91 (38.9)	81 (38.0)	
pT4	62 (26.5)	64 (30.0)	
Missing	1 (0.4)	0	
pN stage, *n* (%)			
pN0	87 (37.2)	83 (39.0)	0.90
pN1	47 (20.1)	37 (17.4)	
pN2	49 (20.9)	44 (20.7)	
pN3	51 (21.8)	49 (23.0)	
Number of lymph nodes, *mean (range)*	18 (2-73)	29 (4-106)	<0.001
TNM (7th edition), *n* (%)			
Stage I	60 (25.6)	52 (24.4)	0.98
Stage II	69 (29.5)	62 (29.1)	
Stage III	98 (41.9)	92 (43.2)	
Stage IV	7 (3.0)	7 (3.3)	

Supplementary Table S1A. Clinicopathological characteristics of the 447 study patients per treatment arm of the D1/D2 trial.

The histopathological variables are determined at central pathology review.

[a]*P*-values are derived from Fisher's exact tests between the three groups. ANOVA was used for continuous variables age and number of lymph nodes.

[b]Excluding those with missing data.

CRITICS trial			
**Characteristic**	**Postoperative chemotherapy (*n* = 226)**	**Postop. chemoradiotherapy (*n* = 228)**	***P* value[a]**
Age at diagnosis (year)			
Median age (IQR)	64 (54-69)	63 (56-69)	0.66
Sex, *n* (%)			
Male	150 (66.4)	159 (69.7)	0.48
Female	76 (33.6)	69 (30.3)	
Tumor localization, *n* (%)			
GE-junction	41 (18.1)	36 (15.8)	0.77
Proximal	39 (17.3)	47 (20.6)	
Middle	71 (31.4)	68 (29.8)	
Distal	75 (33.2)	77 (33.8)	
Lauren classification, *n* (%)			
Diffuse	95 (42.0)	106 (46.5)	0.59
Intestinal	92 (40.7)	83 (36.4)	
Mixed	17 (7.5)	13 (5.7)	
Other	22 (9.7)	26 (11.4)	
ypT stage[c], *n* (%)			
pT1	24 (10.6)	21 (9.2)	0.32[b]
pT2	25 (11.1)	28 (12.3)	
pT3	93 (41.2)	84 (36.8)	
pT4	37 (16.4)	53 (23.2)	
Missing / No resection	47 (20.8)	42 (18.4)	
ypN stage[c], *n* (%)			
pN0	82 (36.3)	88 (38.6)	0.67[b]
pN1	36 (15.9)	29 (12.7)	
pN2	38 (16.8)	40 (17.5)	
pN3	29 (12.8)	36 (15.8)	
Missing / No resection	41 (18.1)	35 (15.4)	
Number of lymph nodes, *mean (range)*	20 (1-66)	19 (1-56)	0.08[b]
yTNM (7th edition), *n* (%)			
Stage 0 / pCR	6 (2.7)	7 (3.1)	0.88[b]
Stage I	35 (15.5)	37 (16.2)	
Stage II	73 (32.3)	69 (30.3)	
Stage III	70 (31.0)	80 (35.1)	
No resection	40 (17.7)	35 (15.4)	
Missing	2 (0.9)	0	
Histopathological response (Mandard), *n* (%)			0.03[b]
Good (TRG 1+2)	20 (8.8)	30 (13.2)	
Moderate (TRG 3)	31 (13.7)	45 (19.7)	
Poor (TRG 4+5 + progression)	158 (69.9)	132 (57.9)	
Unknown	17 (7.5)	21 (9.2)	

Supplementary Table S1B. Clinicopathological characteristics of the 454 study patients per treatment arm of the CRITICS trial.

The histopathological variables are determined at central pathology review.

[a]*P*-values are derived from Fisher's exact tests between the three groups. ANOVA was used for continuous variables age and number of lymph nodes.

[b]Excluding those with missing data.

[c]yp denotes the T, N, and TNM stages after neoadjuvant chemotherapy and surgery in the CRITICS trial.

	CRITICS trial		
	**Biopsy with any amount of mucinous lakes, *n* (%)**	**Biopsy without mucinous lakes, *n* (%)**	***P*-value**
**Percentage of mucinous lakes in resection specimen**			
1-25% (*n* = 5)	2 (40.0)	3 (60.0)	0.07
26-50% (*n* = 6)	4 (66.7)	2 (33.3)	
51-75% (*n* = 5)	3 (60.0)	2 (40.0)	
76-100% (*n* = 10)	10 (100.0)	0	

Supplementary Table S2A. Correlation of mucinous lakes found in pre-treatment biopsies and resection specimens in the CRITICS trial.

The selection is based on all tumors in the CRITICS trial, of which both biopsies and resection specimens were available, and of which mucinous lakes were found in resection specimens.

	MSI-high in the CRITICS trial	
	**Biopsy with any amount of mucinous lakes (*n* = 8)**	**Biopsy without mucinous lakes (*n* = 19)**
**Tumor regression grade**		
TRG 1+2 (*n* = 2)	2	0
TRG 3 (*n* = 1)	0	1
TRG 4+5 + progression (*n* = 24)	6	18

Supplementary Table S2B. Correlation of mucinous lakes found in pre-treatment biopsies and tumor regression grade of MSI-high tumors in the CRITICS trial.

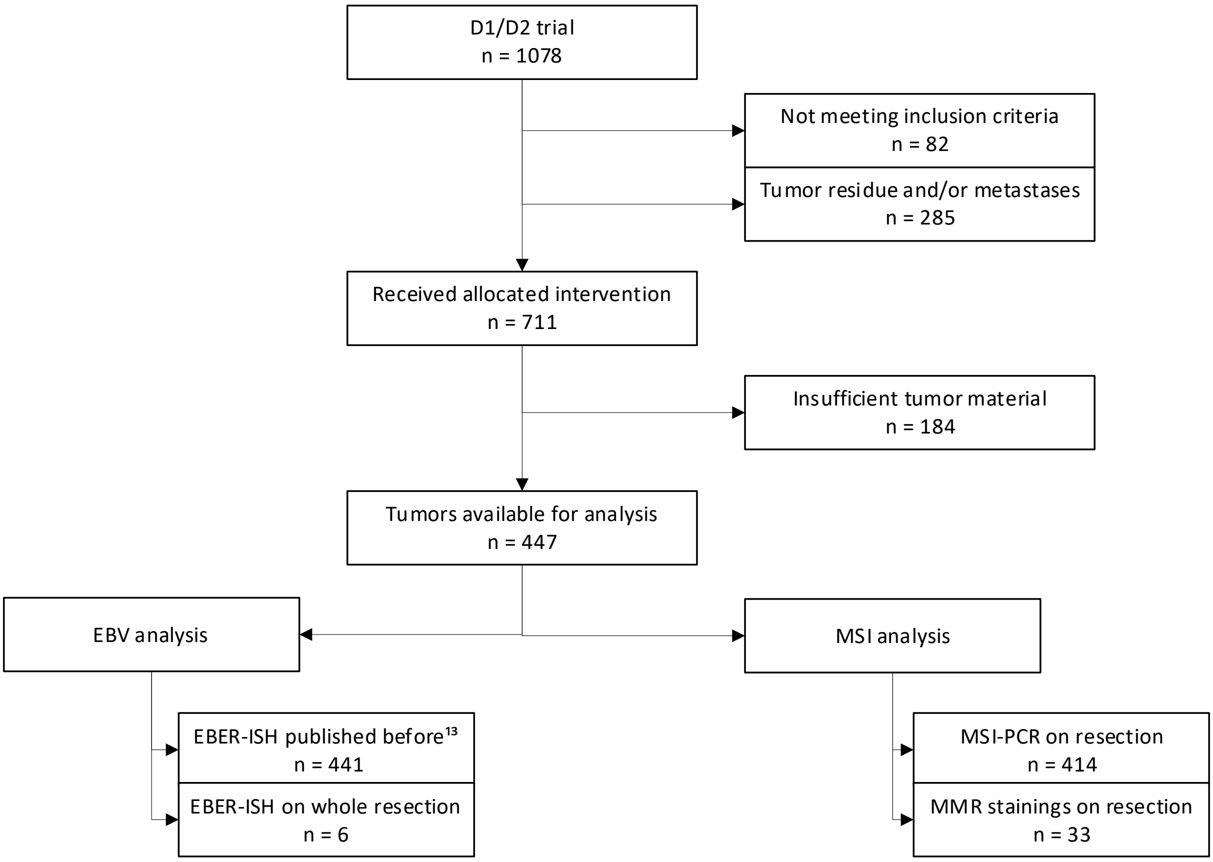

Supplementary Figure S1A. Flowchart of tumor material from patients In the D1/D2 trial used for EBV and MSI analyses.

Abbreviations: EBV, Epstein-Barr virus; MSI, microsatellite instability; EBER-ISH, EBV-encoded RNA in situ hybridization; MMR, mismatch repair.

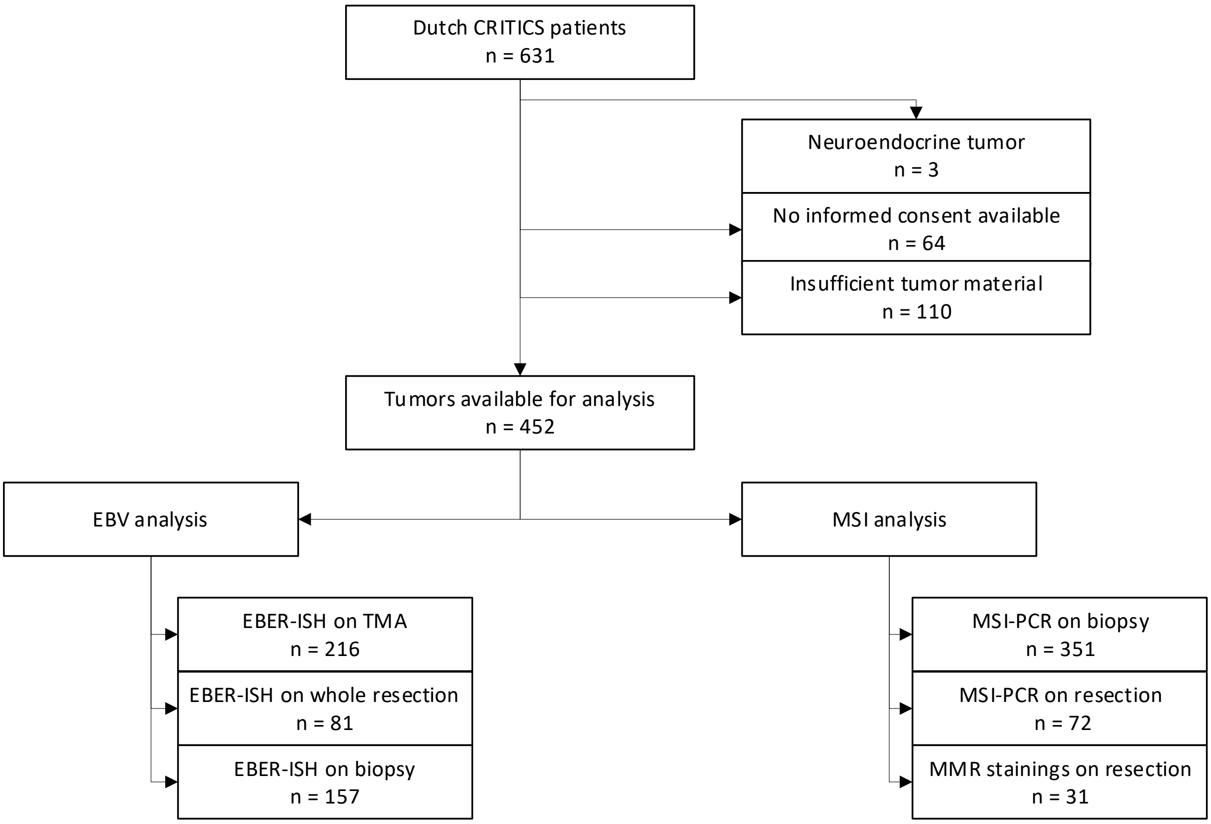

Supplementary Figure S1B. Flowchart of tumor material from patients In the CRITICS trial used for EBV and MSI analyses.

Abbreviations: EBV, Epstein-Barr virus; MSI, microsatellite instability; EBER-ISH, EBV-encoded RNA in situ hybridization; TMA, tissue microarray; MMR, mismatch repair.

(a)

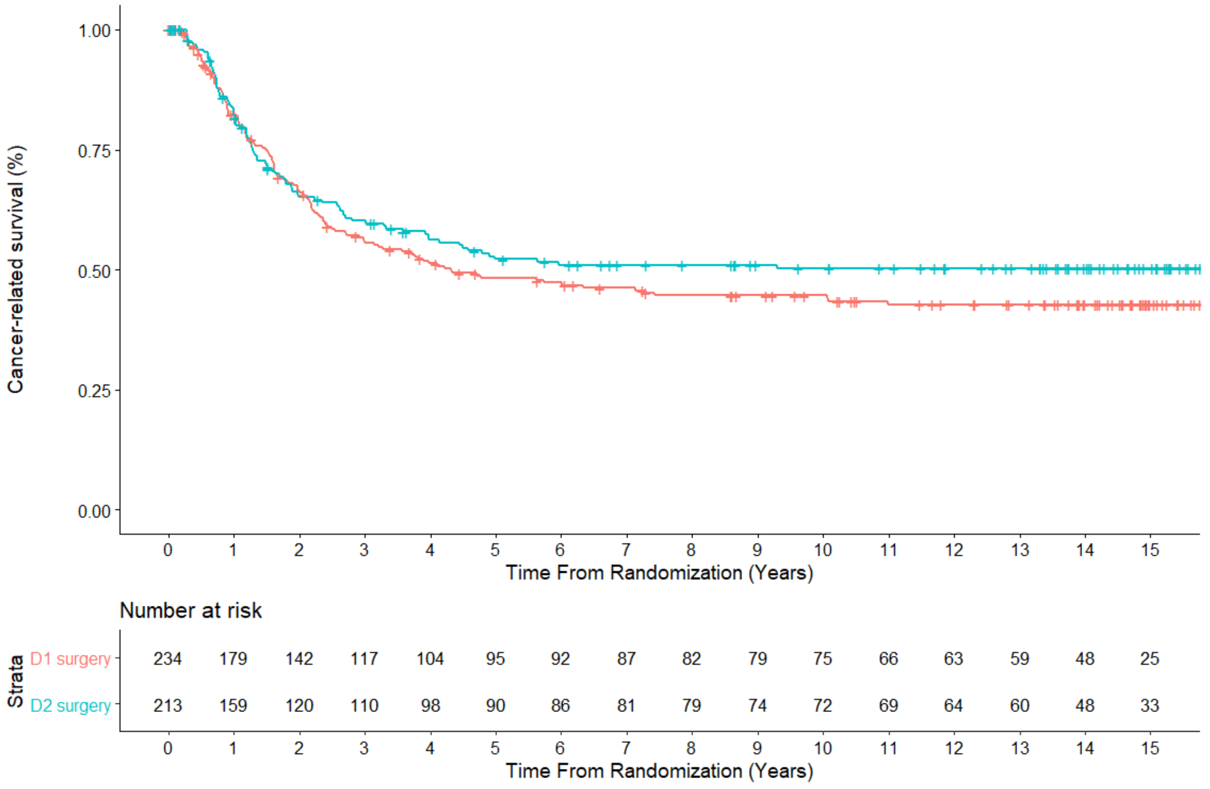
Cancer-related survival (%)
1.00
0.75
0.50
0.25
0.00
0 1 2 3 4 5 6 7 8 9 10 11 12 13 14 15
Time From Randomization (Years)
Number at risk
Strata
D1 surgery 234 179 142 117 104 95 92 87 82 79 75 66 63 59 48 25
D2 surgery 213 159 120 110 98 90 86 81 79 74 72 69 64 60 48 33
0 1 2 3 4 5 6 7 8 9 10 11 12 13 14 15
Time From Randomization (Years)

(b)

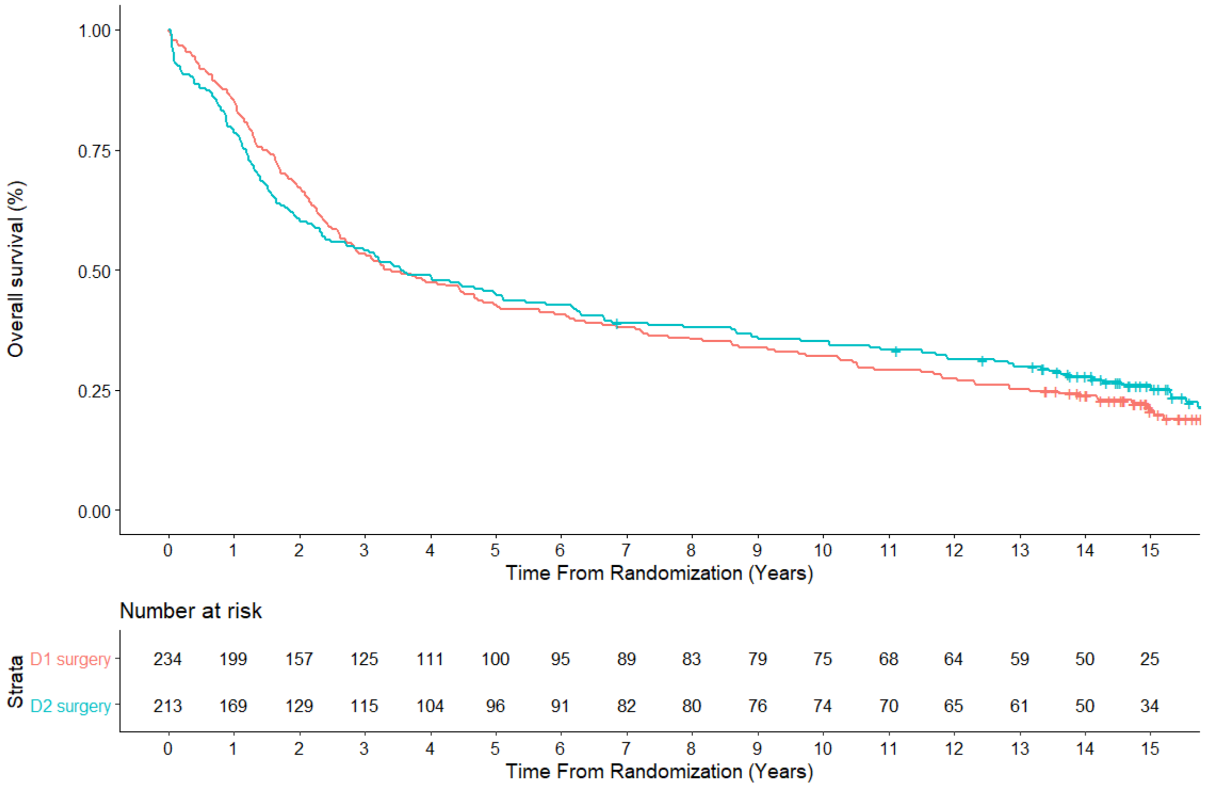
Overall survival (%)
1.00
0.75
0.50
0.25
0.00
0 1 2 3 4 5 6 7 8 9 10 11 12 13 14 15
Time From Randomization (Years)
Number at risk
Strata
D1 surgery 234 199 157 125 111 100 95 89 83 79 75 68 64 59 50 25
D2 surgery 213 169 129 115 104 96 91 82 80 76 74 70 65 61 50 34
0 1 2 3 4 5 6 7 8 9 10 11 12 13 14 15
Time From Randomization (Years)

Supplementary Figure S2A. (a) Cancer-related and (b) overall survival since randomization in the 447 patients per treatment arm of the D1/D2 trial.

(a) Five-year cancer-related survival was 48.3% for D1, and 52.2% for D2 surgery. The hazard ratio was 0.85 (95%CI=0.65-1.12, *P*=.25) for D2 vs D1 surgery. (b) Five-year overall survival was 42.7% for D1, and 45.1% for D2 surgery. The hazard ratio was 0.96 (95%CI=0.78-1.19, *P*=.72) for D2 vs D1 surgery.

(a)

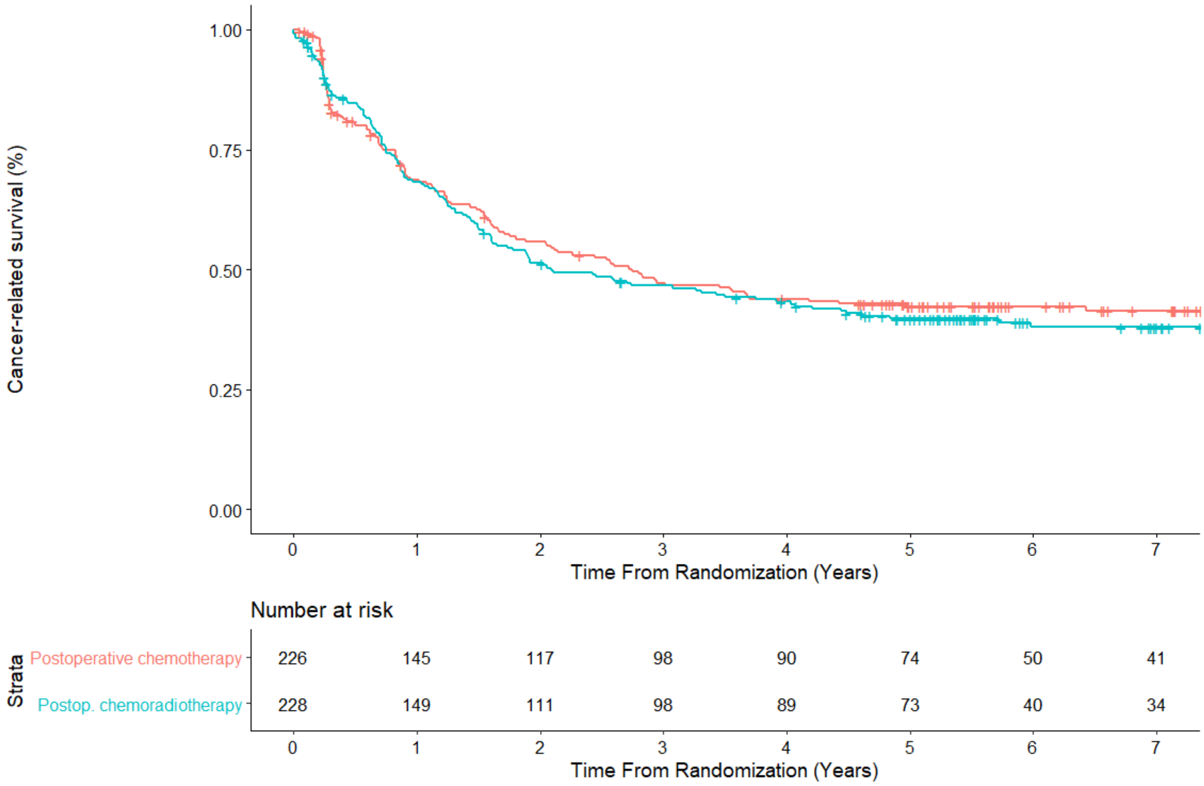
Cancer-related survival (%)
1.00
0.75
0.50
0.25
0.00
0 1 2 3 4 5 6 7
Time From Randomization (Years)
Number at risk
Strata
Postoperative chemotherapy 226 145 117 98 90 74 50 41
Postop. chemoradiotherapy 228 149 111 98 89 73 40 34
0 1 2 3 4 5 6 7
Time From Randomization (Years)

(b)

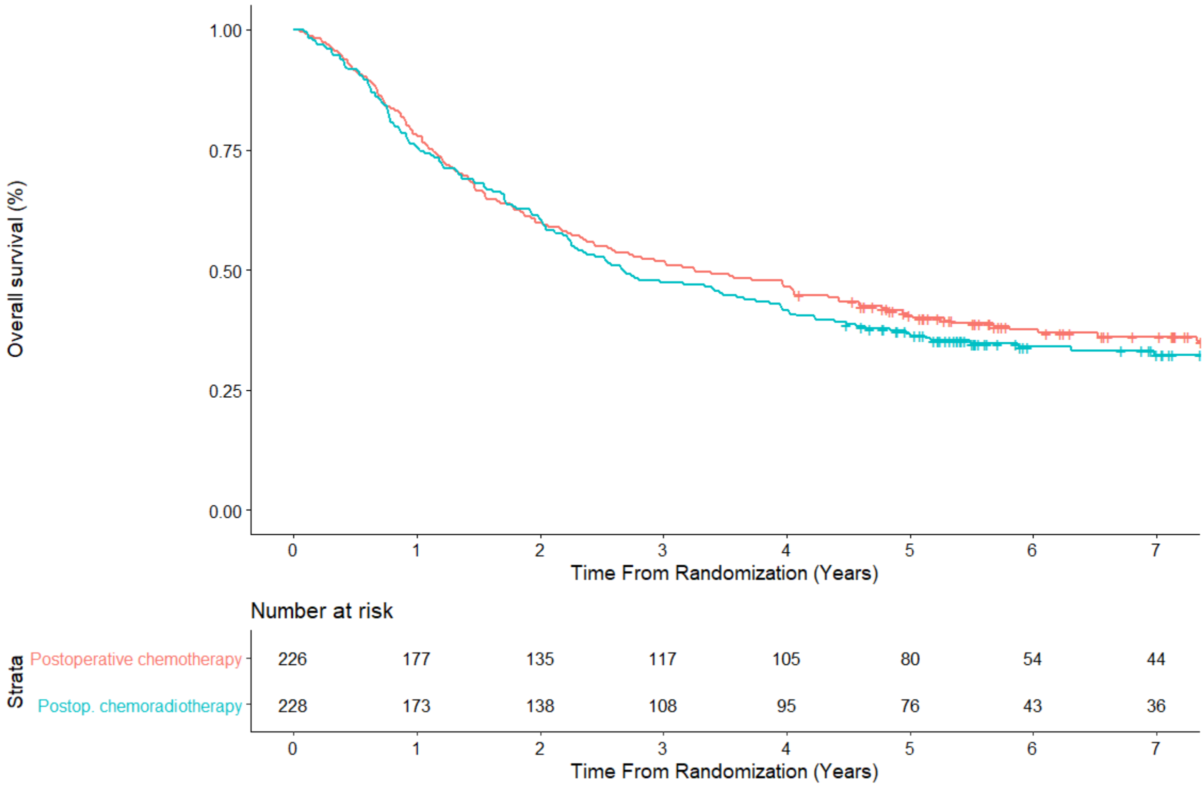
Overall survival (%)
1.00
0.75
0.50
0.25
0.00
0 1 2 3 4 5 6 7
Time From Randomization (Years)
Number at risk
Strata
Postoperative chemotherapy 226 177 135 117 105 80 54 44
Postop. chemoradiotherapy 228 173 138 108 95 76 43 36
0 1 2 3 4 5 6 7
Time From Randomization (Years)

Supplementary Figure S2B. (a) Cancer-related and (b) overall survival since randomization in the 454 study patients per treatment arm of the CRITICS trial.

Cancer-related and overall survival since randomization of the 447 study patients per treatment arm of the D1/D2 trial.

(a) Five-year cancer-related survival was 42.3% for postoperative chemotherapy, and 39.8% for chemoradiotherapy. The hazard ratio was 1.07 (95%CI=0.84-1.37, *P*=.56) for postoperative chemoradiotherapy vs chemotherapy. (b) Five-year overall survival was 40.5% for postoperative chemotherapy, and 36.8% for chemoradiotherapy. The hazard ratio was 1.11 (95%CI=0.88-1.39, *P*=.37) for postoperative chemoradiotherapy vs chemotherapy.

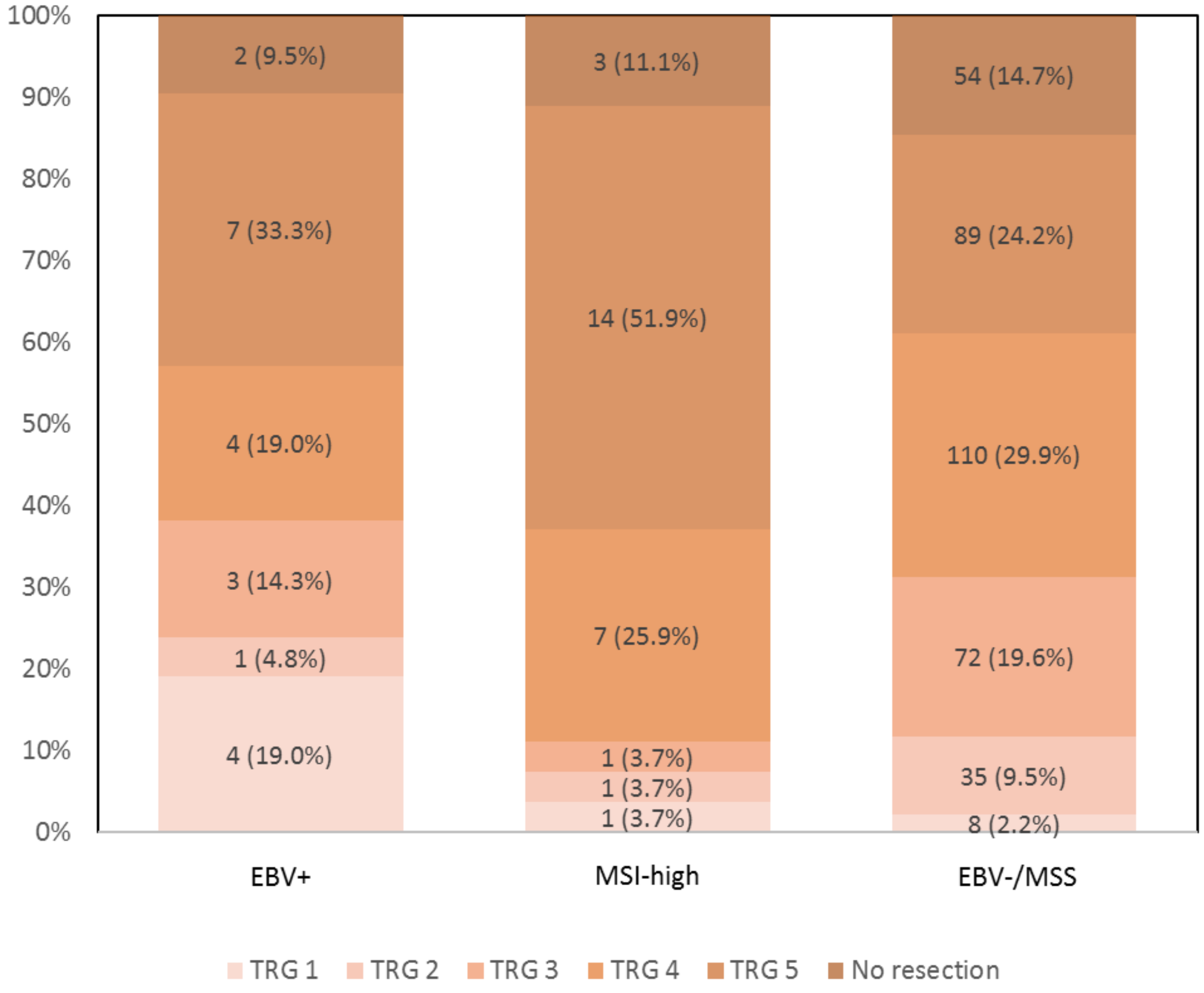

Supplementary Figure S3. Histopathological response according to Mandard per molecular subgroup in the CRITICS trial.

Abbreviations: EBV+, Epstein-Barr virus positive; MSI-high, microsatellite instable; EBV-/MSS, Epstein-Barr virus negative and microsatellite stable.

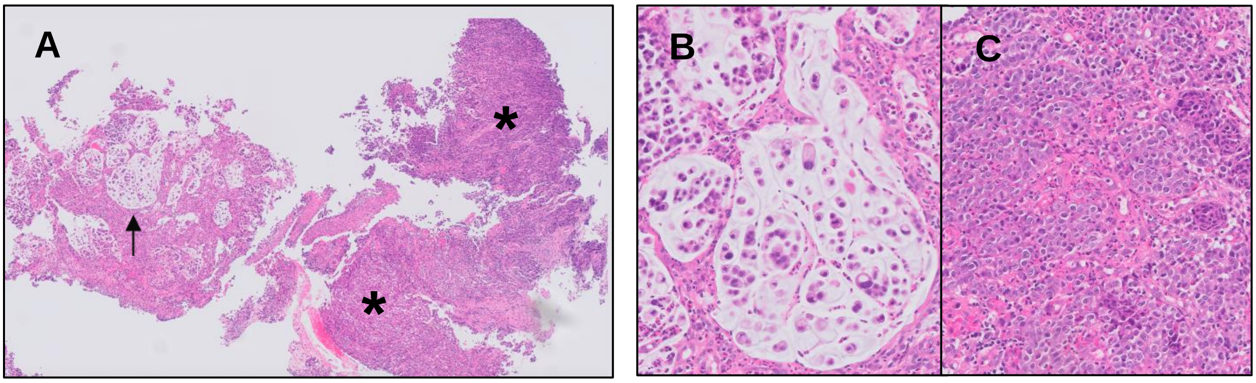

Supplementary Figure S4. Biopsy specimen of a MSI-high carcinoma with mucinous differentiation.

(a) Microscopic image of a diagnostic biopsy specimen of a MSI-high mucinous adenocarcinoma with complete histopathological response after neoadjuvant chemotherapy and surgery showing areas with mucinous differentiation (arrow) and solid growth pattern (asterisk). (b) Detail of a mucinous area. (c) Detail of solid growth pattern.
